# On Modeling Missing Data of an Incomplete Design in the CFA Framework

**DOI:** 10.3389/fpsyg.2020.581709

**Published:** 2020-12-03

**Authors:** Karl Schweizer, Andreas Gold, Dorothea Krampen, Tengfei Wang

**Affiliations:** ^1^Faculty of Psychology and Sports Sciences, Institute of Psychology, Goethe University Frankfurt, Frankfurt, Germany; ^2^Department of Psychology and Behavioral Sciences, Zhejiang University, Hangzhou, China

**Keywords:** missing data, incomplete design, structural investigation, confirmatory factor analysis, quantitative methods, planned missing data design

## Abstract

The paper reports an investigation on whether valid results can be achieved in analyzing the structure of datasets although a large percentage of data is missing without replacement. Two types of confirmatory factor analysis (CFA) models were employed for this purpose: the missing data CFA model with an additional latent variable for representing the missing data and the semi-hierarchical CFA model that also includes the additional latent variable and reflects the hierarchical structure assumed to underlie the data. Whereas, the missing data CFA model assumes that the model is equally valid for all participants, the semi-hierarchical CFA model is implicitly specified differently for subgroups of participants with and without omissions. The comparison of these models with the regular one-factor model in investigating simulated binary data revealed that the modeling of missing data prevented negative effects of missing data on model fit. The investigation of the accuracy in estimating the factor loadings yielded the best results for the semi-hierarchical CFA model. The average estimated factor loadings for items with and without omissions showed the expected equal sizes. But even this model tended to underestimate the expected values.

Datasets originating from empirical research can show missing data because of various reasons. On reason is incompleteness that is intentional as, for example, can be found in datasets based on *planned missing data designs*. Such designs are proposed for reducing the costs of empirical research and to take advantage of a possible increase of validity due to a reduced burden on the participants (Rhemtulla and Hancock, 2016; Rioux et al., 2020). Although incompleteness of a dataset means lack of information that would otherwise be available, this does not mean that results obtained in investigating such data are without value. Missing data can, for example, be replaced by the full information maximum likelihood estimation method (Enders, 2013) or by using the multiple imputation approach (van Buuren, 2018). Such replacement enables the investigation of data by all kinds of analysis methods. However, such replacement implies the danger of distortion of structure. For example, the consistency of data can get overly increased, as can be observed when replacing missing data on the basis of participants' previous performance without considering random influences. Therefore, in the present paper we explore the possibility to investigate incomplete datasets without replacement. This is accomplished for binary data requiring the investigation of structure. The major characteristic of the considered modeling approach is that missing data are modeled instead of replaced. This means that systematic variation that originates from the lack of data is captured by a latent variable of the measurement model so that it no longer impairs the estimation of factor loadings and model fit.

This modeling approach is inspired by recent work on missing data because of a time limit in testing. Such a time limit is likely to enable some participants to provide responses to all items but also prevents other participants from completing all of them (Partchev et al., 2013; Schweizer et al., 2020b). The participants showing missing data because of the time limit usually differ widely among each other regarding the number of not-reached items that suggests dependency on an attribute, which the participants have in common. There is reason for assuming that processing speed determines the number of items that can be reached by the participant, as is suggested by the rational of speed testing (Lord and Novick, 1968).

Processing speed also determines the frequency distribution of missing data that characterizes the dataset. Following Rhemtulla and Hancock (2016), we consider it as a specific type of *missingness*. Because of the time limit the distribution of processing speed is mapped into the (expected) frequency distribution of the missing data. Information on this distribution can be used for establishing an additional latent variable as part of a confirmatory factor analysis (CFA) model that reflects the type of missingness of data directly and in this case processing speed indirectly. But it is the expected frequency distribution that is considered for this purpose since the observed frequency distribution can be assumed to reflect other influences additionally. Such a model enables structural investigations without the replacement of missing data (Schweizer et al., 2019).

Since the modeling approach only requires information on the frequency distribution of the missing data, this approach can be transferred to other datasets showing other types of missingness. For example, it can be transferred to intentionally incomplete datasets because of a planned missing data design. Missing data originating from such a design have a special quality: they are missing completely at random (Rhemtulla and Hancock, 2016). Missing data showing this quality do not differ in a systematic way from the available data. In investigations making use of such a design the researcher determines the missing data and, therefore, the frequency distribution of the missing data is known.

## The Modeling of Missing Data With a Two-Factor Model

Other than the regular CFA model (Graham, 2006), the measurement model for investigating the structure of a dataset with missing data includes two latent variables instead of one. There is one latent variable that represents the source of responding that is usually an ability or trait. We symbolize it by ξ_genuine_. In factor analysis as data decomposition according to the model of the covariance matrix (Jöreskog, 1970), this latent variable is expected to capture the systematic variation that originates from the ability or trait of interest. The other latent variable aims at systematic variation that originates from missing data. A regular pattern of missing data can exert a systematic influence on the set or a subset of covariances that becomes apparent as systematic variation in factor analysis. This kind of systematic variation needs to be captured by the other latent variable for achieving good model fit. We symbolize this latent variable by ξ_missing_. Equation (1) provides the formal description of the measurement model for investigating datasets with missing data:

(1) $$\begin{array}{l} \text{x}=\lambda_{\text{genuine}}\xi_{\text{genuine}}+\lambda_{\text{missing}}\xi_{\text{missing}}+\delta \end{array}$$

where **x** is the *p* × 1 vector of manifest variables, **λ**_genuine_ and **λ**_missing_ the *p* × 1 vectors of factor loadings on the genuine factor, ξ_genuine_, and the factor of the missing data, ξ_missing_, respectively, and **δ** the *p* × 1 vector of error variables. We refer to it as *missing data CFA model*.

An important prerequisite for modeling missing data is information on their frequency distribution. In modeling missing data due to a time limit in testing, this information is provided by the source of the missing data that is latent processing speed. In missing data due to an incomplete design, the information on the frequency distribution of missing data is available from the design. For each item *i* (*i* = 1, …, *p*) the expected number of missing data, *n*_*i*_, can be ascertained from the design. Since only the information on the relationships between the frequencies for the items counts in factor analysis, adjusting the frequencies to the typical sizes of factor loadings is a modification that does not influence the outcome but can support the estimation process. Adjustment can be achieved by multiplication with constant *c* (>0). Making use of this information for the establishment of the corresponding factor requires the integration of the adjusted frequencies into the *p* × 1 vector of factor loadings, **λ**_missing_:

(2) $$\begin{array}{l} \lambda_{\text{missing}}=\left[\begin{array}{c} c\times n_{\text{missing\_}1}\\ c\times n_{\text{missing\_}2}\\ .\\ .\\ c\times n_{\text{missing\_}p} \end{array}\right] \end{array}$$

where *n*_missing_*i*_ is the number of participants who are expected not to provide a response to the *i*th item. In a systematically incomplete design a number of items may show no missing data so that a larger number of entries of **λ**_missing_ may be set to zero.

The constraint of the entries of **λ**_missing_, as is suggested by Equation (2), necessitates the freeing of the associated variance parameter, φ_missing_, of the model of the *p* × *p* covariance matrix, **Σ**, for estimation. Estimation is accomplished as minimization of the difference between **Σ** and the *p* × *p* empirical covariance matrix, **S**, using discrepancy function F:

(3) $$\begin{array}{l} \text{F}\left[\text{S},\Sigma \right]. \end{array}$$

The estimate of φ_missing_ reflects the amount of systematic variation that is in line with the profile of constraints included in **λ**_missing_.

The constraint of the factor loadings of **λ**_missing_ does not imply any restriction to the factor loadings on ξ_genuine_. This means that the factor loadings included in **λ**_genuine_ can be defined as free or constrained parameters.

## The Modeling of the Hierarchical Structure

This section introduces the semi-hierarchical CFA model that is another model suitable for investigating data with omissions because of a time limit in testing (Schweizer et al., 2020a). This model shows a property, which the missing data CFA model lacks. The missing data CFA model implicitly assumes to be valid for all participants, i.e., everyone's performance is influenced by the latent variables of the model. This assumption is incorrect in data with omissions since in some participants only ξ_genuine_ contributes to the outcome and in other participants also ξ_missing_. The semi-hierarchical model takes this difference into account.

In the following, the adaptation of the missing data model to data showing a hierarchical structure is described. Assuming a hierarchical structure underlying data is suggested for samples composed of subsamples that differ according to the source that mainly determines responding (Burnstein, 1980; Preacher et al., 2016). Such a structure applies, for example, if in one subsample a specific ability (other than processing speed) mainly determines performance and in the other subsample mainly processing speed. The two sources give rise to a hierarchy with two levels. There is the first level that comprises all participants and the second level that includes the subsamples (Raudenbush and Bryk, 2002; Snijders and Bosker, 2012). The existence of such a hierarchical structure provides the opportunity to consider specific regression weights or factor loadings for each one of the two subsamples.

For the purpose of considering the hierarchical structure it is necessary to shift the focus from the set of items to the individual item. Given the focus shifted to individual item *i* (*i* = 1, …, *p*), the sample of participants, C_*i*_, can be subdivided into the subsample of participants who have the opportunity to provide a response, C_with_response_option_*i*_, and the subsample of participants without such an opportunity, C_without_response_option_*i*_:

(4) $$\begin{array}{l} {\text{C}}_{i}=\left\{{\text{C}}_{\text{with\_response\_option\_}i},{\text{C}}_{\text{without\_response\_option\_}i}\right\}. \end{array}$$

For Equation (1) this means the shift from **x** to *x*_*i*_:

(5) $$\begin{array}{l} x_{i}=\lambda_{\text{genuine\_}i}\xi_{\text{genuine}}+\lambda_{\text{missing\_}i}\xi_{\text{missing}}+\delta_{i}. \end{array}$$

The new focus enables the consideration of the characteristics of the subsamples. It becomes apparent that only ξ_genuine_ is active in subsample C_with_response_option_*i*_ and only ξ_missing_ in subsample C_without_response_option_*i*_:

(6) $$\begin{array}{l} x_{\text{with\_response\_option\_}i}=\lambda_{\text{genuine\_}i}\xi_{\text{genuine}}+0\times \xi_{\text{missing}}+\delta_{i} \end{array}$$

and

(7) $$\begin{array}{l} x_{\text{without\_response\_option\_}i}=0\times \xi_{\text{genuine}}+\lambda_{\text{missing\_}i}\xi_{\text{missing}}+\delta_{i} \end{array}$$

The integration of the specificities of the second-level structure into a common equation can be achieved by the adaptation of the contributions of the latent variables to the sizes of the subsamples. It is realized by the use of subsample-specific weights: *w*_with_response_option_*i*_ for participants with the opportunity to respond and *w*_without_response_option_*i*_ for the other participants:

(8) $$\begin{array}{l} x_{i}=w_{\text{with\_response\_option\_}i}\lambda_{\text{genuine\_}i}\xi_{\text{genuine}}\\ \;+w_{\text{without\_response\_option\_}i}\lambda_{\text{missing\_}i}\xi_{\text{missing}}+\delta_{i}. \end{array}$$

The weights, *w*_with_response_option_*i*_ and *w*_without_response_option_*i*_, moderate the effects of ξ_genuine_ and ξ_missing_ on *x*_*i*_. These weights relate the numbers of participants included in C_with_response_option_*i*_, *n*_with_response_option_*i*_, and in C_without_response_option_*i*_, *n*_without_response_option_*i*_, to the number of participants of the complete sample, *N*:

(9) $$\begin{array}{l} w_{\text{with\_response\_option\_}i}=\sqrt{n_{\text{with\_response\_option\_}i}/N} \end{array}$$

and

(10) $$\begin{array}{l} w_{\text{without\_response\_option\_}i}=\sqrt{n_{\text{without\_response\_option\_}i}/N} \end{array}$$

The square root is additionally computed because in the course of data analysis according to the model of the covariance matrix the ratio that is added as multiplier to the factor loading is squared (Jöreskog, 1970). Otherwise the computation of the square root is not necessary.

In order to regain the status of measurement model, the focus must be shifted back from the individual items to the set of items, i.e., from the individual variable to the vector. It requires the integration of the weights *w*_with_response_option_*i*_ and *w*_without_response_option_*i*_ into the main diagonals of the *p* × *p* diagonal weight matrices **W**_with_response_option_ and **W**_without_response_option_. After inserting these weight matrices in Equation (1) the final measurement model for incomplete data is achieved:

(11) $$\begin{array}{l} \text{x}={\text{W}}_{\text{with\_response\_option}}\lambda_{\text{genuine}}\xi_{\text{genuine}}\\ \;+{\text{W}}_{\text{without\_response\_option}}\lambda_{\text{mising}}\xi_{\text{missing}}+\delta . \end{array}$$

Such a model is referred to as *semi-hierarchical CFA model* (Schweizer et al., 2020a).

**Figure 1:**
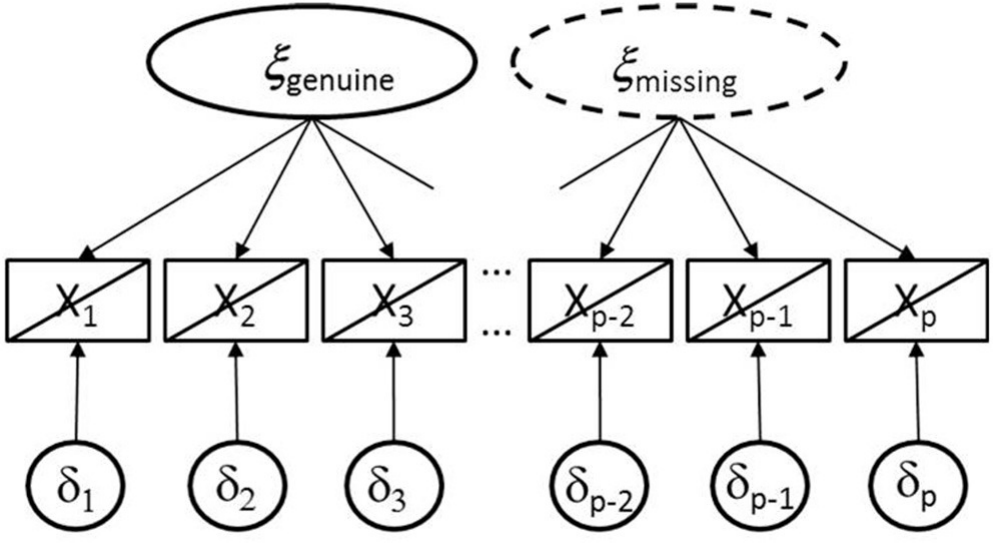
Illustration of the semi-hierarchical model including genuine and missing data latent variables with a diagonal line assigned to rectangles of manifest variable for signifying that in some participants both latent variables are active whereas in the others only the first one. The ellipse printed as dashed line signifies that this latent variable is only active in a subsample.

Figure 1 provides an illustration of the semi-hierarchical CFA model. The two ellipses demonstrate the two latent variables. The ellipse printed as solid line characterizes ξ_genuine_ that can be assumed to be active in all participants whereas the ellipse printed as dashed line refers to ξ_missing_ that is expected to be active in participants with omissions only. Furthermore, the diagonal lines assigned to the rectangles of manifest variables signify that there are subsamples with different underlying structures.

**Figure 2:**
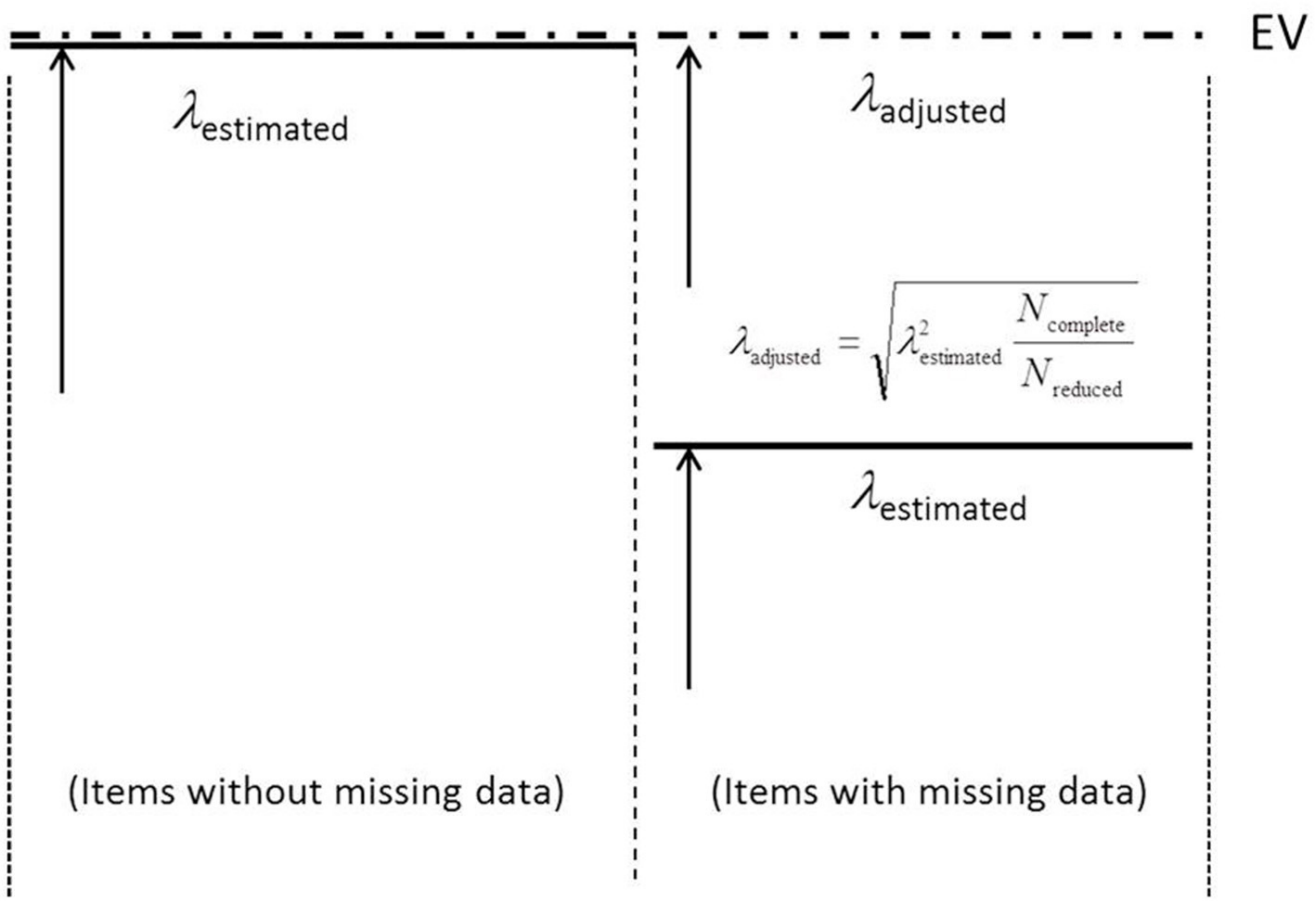
Illustration of the adjustment step for factor loadings estimated by the semi-hierarchical CFA model. EV, Expected Value.

The weights of Equation (11) enable the second level of the hierarchical structure to contribute to model fit. However, in an investigation with the focus on the accuracy of estimating the factor loadings, weights may be considered as disadvantageous. Because of the weights that vary between zero and one (see Equations 9 and 10) the factor loadings on ξ_genuine_ show a smaller size than otherwise. This is illustrated for missing data because of an incomplete design by Figure 2.

The solid horizontal lines represent the estimated factor loadings on ξ_genuine_ and the dashed line the expected factor loadings. This line is labeled as EV (abbreviation of expected value) in Figure 2. In items with no missing data, the weight associated with ξ_genuine_ is one and the estimates of the factor loadings should closely correspond to the expected sizes (see left-hand part of Figure 2). In contrast, in the case of a weight smaller than one the factor loadings should show a reduced size (see right-hand part of Figure 2). They should show a reduction that reflects the amount of missing data.

In order to eliminate the influence of missing data on the size of these factor loadings, an adjustment is recommended that is described by Equation (12):

(12) $$\begin{array}{l} \lambda_{\text{adjusted\_}i}={\left(\lambda_{\text{estimated\_}i}^{2}\frac{N}{n_{\text{with\_response\_option\_}i}}\right)}^{1/2}. \end{array}$$

This adjustment reverses the effect of the weight attached to the factor loading on ξ_genuine_. Note: This adjustment has to follow the estimation of the parameters. If there is additional standardization of the factor loadings, it is also important that this adjustment precedes the standardization according to the items.

## The Input to CFA

This section explains how the information on the missing data is integrated into the covariances that are input to CFA. We proceed from the assumption that binary data have to be investigated. This assumption enables the consideration of the probability-based covariance coefficient, cov(*X*_*i*_, *X*_*j*_), for binary variables *X*_*i*_ and *X*_*j*_ (*i, j* = 1, …, *p*):

(13) $$\begin{array}{l} \mathrm{cov}\left(X_{i},X_{j}\right)=\mathrm{Pr}\left(X_{l}=1\wedge X_{j}=1\right)-\mathrm{Pr}\left(X_{i}=1\right)\mathrm{Pr}\left(X_{j}=1\right), \end{array}$$

where 1 serves as the code for the correct response. This coefficient requires the computation of the difference between the probability of co-occurrences of 1 in both variables and the product of two other probabilities. Only information on the correct responses is necessary for achieving the probability-based covariance. It is not necessary to distinguish between incorrect responses and missing responses. The covariance is similar to a pre-stage that is reached in computing the Phi coefficient.

The probabilities included in Equation (13) reflect the influence of missing data. In order to demonstrate this statement, we assume that in the complete dataset the probability of a correct response depends on ξ_genuine_:

(14) $$\begin{array}{l} \mathrm{Pr}\left(X_{i}=1\right)=\mathrm{Pr}\left(X_{i}=1|\xi_{\text{genuine}}\right). \end{array}$$

If there are missing data, Equation (14) is not true. It only applies to the subsample showing complete data. This restriction can be taken into consideration by the multiplication of the right-hand term of Equation (14) with the probability that *X*_*i*_ is not missing. Replacing the probability that *X*_*i*_ is not missing by one minus the probability of *X*_*i*_ being missing, Pr(*X*_*i*_ is missing), finally gives

(15) $$\begin{array}{l} \mathrm{Pr}\left(X_{i}=1\right)=\mathrm{Pr}\left(X_{i}=1{|\xi }_{\text{genuine}}\right)\times \left[1-\mathrm{Pr}\left(X_{i}\text{ is missing}\right)\right]. \end{array}$$

From Equation (15) it is apparent that there is a systematic influence of missing data on the probabilities giving rise to the probability-based covariance. The probability-based covariances for datasets including missing data can be expected to differ in a systematic way from the probability-based covariances for complete datasets.

## A Simulation Study

The simulation study served the evaluation of the two described CFA models, the missing data CFA model and the semi-hierarchical CFA model, for investigating the structure of data generated according to an incomplete design. Up to 25% of the entries of regular datasets of structured random data were turned into missing data for this investigation. Furthermore, there was variation of the number of columns showing missing data. The investigation focused on model fit and accuracy in estimating the sizes of factor loadings. The performance of the one-factor CFA model served as comparison level.

## Method

Five-hundred 500 × 20 matrices of random data were generated and modified to show different missing data conditions. The underlying one-dimensional structure was achieved by the procedure described by Jöreskog and Sörbom (2001). This procedure required the preparation of a relational pattern. The diagonal entries of this relational pattern were 1.00. Off-diagonal entries were selected to be reproducible by a one-factor model with factor loadings of 0.35.

The generated data included in the 500 × 20 matrices were continuous and normally distributed. For achieving binary data, the continuous and normally distributed data were dichotomized into zeros and ones. The numbers included in the columns of the matrices were dichotomized such that the probability of one (=correct response) was 0.95 in the first column and 0.5 in the last column. The probabilities assigned to the other columns linearly decreased from the first to last one.

The incompleteness of the design was achieved by eliminating entries of either four (columns 17–20), seven (columns 14–20) or 10 (columns 11–20) of the total of 20 columns of the generated data matrices. We selected columns from the end of the sequence of columns because omissions seemed to be a bit more likely in the end of the sequence of columns than in the beginning. But this choice did not imply that we expected special results for these subsets of columns. Furthermore, the percentages of eliminated entries were varied. Each complete data matrix was turned into five incomplete matrices by eliminating 10, 20, 30, 40 and 50% of the entries of the selected columns. While the rows for eliminating entries were selected according to a quasi-random scheme, the columns selected for the elimination of entries were kept constant.

Probability-based covariances (see Equation 13) served as input to confirmatory factor analysis. Additionally, a link transformation was performed when modeling the data in order to overcome the difference between the binomially distributed data and the latent variables of the model following the normal distribution (McCullagh and Nelder, 1985; Schweizer et al., 2015).

The factor loadings of the CFA models were constrained. The loadings for manifest variables on the genuine factor were constrained in the manner of generating random data; they were set to equal sizes. The same applied to the loadings on the factor of the missing data with respect to columns showing missing data whereas the other loadings were set to zero. In the semi-hierarchical model the factor loadings additionally received weights according to Equations (9) and (10).

Estimated models included either one or two factors. The one-factor model only included the genuine factor as latent variable and 20 manifest variables. The two-factor models were either specified as missing data CFA model (see Equation 1) or semi-hierarchical CFA model (see Equation 11).

The LISREL software package (Jöreskog and Sörbom, 2006) with maximum likelihood estimation was used. We employed the following fit indices and criteria (in parentheses) for this study: χ^2^, RMSEA (≤0.06), SRMR (≤0.08), CFI (≥0.95), and TLI (≥0.95) (see Hu and Bentler, 1999; DiStefano, 2016). The CFI difference (Cheung and Rensvold, 2002) was used to compare models.

## Results

The statistical investigation aimed at finding out whether there was impairment in either model fit or accuracy due to missing data despite the model-implied provisions regarding missing data. At the same time it was to demonstrate that in the absence of such provisions there were such detrimental effects due to missing data. The results regarding model fit are presented first and the results regarding accuracy subsequently.

### Results Regarding Model Fit

The mean fit statistics observed in investigating datasets with four columns showing between 10 and 50% missing data are presented in Table 1.

**Table 1:** Fit results obtained by the one-factor CFA model, the missing data CFA model and the semi-hierarchical CFA model in investigating data showing different percentages of missing data in items 17–20 (*N*_withoutmissingdata_ = 500).

**Percentage of missing data**	**χ^**2**^**	**df**	**RMSEA**	**SRMR**	**TLI**	**CFI**	**AIC**
**One-factor CFA model applied to complete data**							
0	205.6	189	0.012	0.045	0.953	0.947	247.6
**One-factor CFA model**							
10	239.8	189	0.022	0.048	0.884	0.884	281.8
20	315.3	189	0.036	0.055	0.780	0.781	357.3
30	415.6	189	0.049	0.063	0.689	0.691	457.6
40	527.6	189	0.060	0.070	0.618	0.620	569.6
50	644.2	189	0.069	0.078	0.561	0.564	686.2
**Missing data CFA model**							
10	203.6	188	0.011	0.044	0.960	0.955	247.6
20	203.3	188	0.011	0.044	0.965	0.961	247.3
30	204.4	188	0.011	0.044	0.968	0.965	248.4
40	205.7	188	0.012	0.045	0.970	0.968	249.7
50	207.8	188	0.013	0.045	0.971	0.969	251.8
**Semi-hierarchical CFA model**							
10	204.1	188	0.011	0.044	0.957	0.953	248.1
20	202.5	188	0.011	0.044	0.966	0.961	246.5
30	203.0	188	0.011	0.044	0.970	0.966	247.0
40	203.6	188	0.011	0.044	0.974	0.970	247.6
50	204.6	188	0.012	0.044	0.975	0.972	248.6

The first major part of Table 1 includes the results obtained by the one-factor CFA model, the second major part comprises the results of the missing data CFA model, and the third major part contains the results of the semi-hierarchical CFA model.

The results of the very first row that precedes the major parts of Table 1 were obtained by applying the one-factor CFA model to complete datasets. Since the data were generated to show a one-dimensional underlying structure, this model was expected to fit well in the absence of missing data. The fit results were in line with this expectation except for the CFI statistic. It only indicated marginally good model fit.

The following major part of Table 1 includes the results obtained by the one-factor CFA model. The χ^2^ observed for this model proved to be sensitive for the amount of missing data. It increased from 239.8 to 644.2 when the percentage of missing data was increased from 10 to 50%. RMSEA and SRMR always indicated good or acceptable fit for this model whereas TLI and CFI always indicated poor model fit.

In contrast, the χ^2^ observed for the missing data CFA model and the semi-hierarchical CFA model of the following major parts of Table 1 varied only within a very small range despite the large variation of the amount of missing data. Furthermore, all RMSEA, SRMR, TLI, and CFI statistics signified good model fit. The differences between the statistics for these two two-factor models were minor. In no case a comparison by the CFI difference test yielded a substantial result.

**Figure 3:**
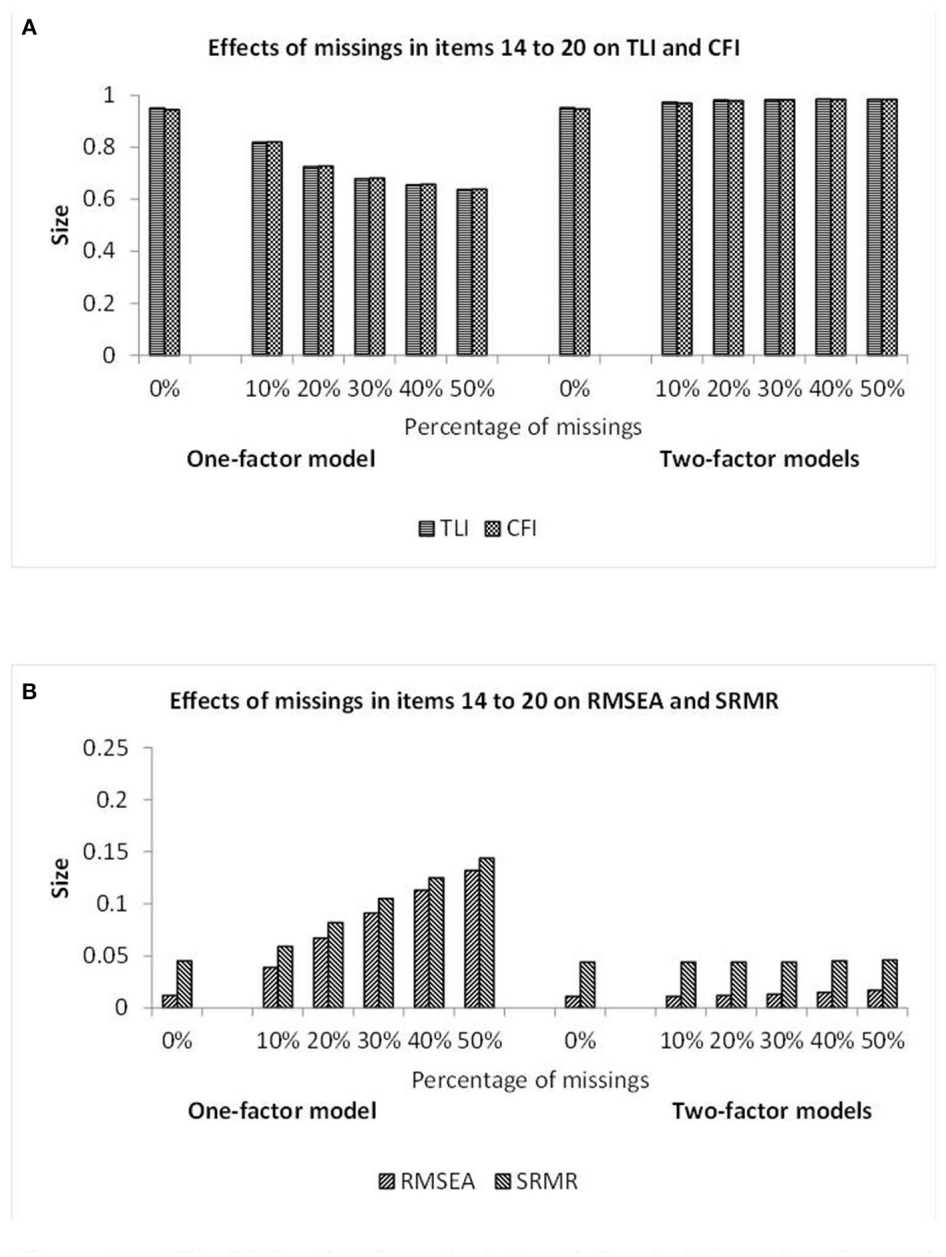
Illustration of the TLI and CFI results **(A)** and also the RMSEA and SRMR results **(B)** observed when investigating the data showing different percentages of missing observations in items 14–20 by one-factor (see first half) and two-factor (see second half) CFA models. Note: The missing data and semi-hierarchical CFA models yielded the virtually same results.

Since quite similar patterns of results were observed for data with missing observations in seven and 10 columns, these results are presented as bars of figures instead of numbers included in tables. Furthermore, since the missing data and semi-hierarchical CFA models yielded the virtually same results, the figures only include bars for what is labeled as two-factor model and applies to both models. Figure 3 provides the fit results for datasets with missing data in seven columns.

According to Figure 3A, the TLI and CFI statistics indicated poor model fit for the one-factor CFA model and good model fit for the other models. The TLI and CFI statistics of the one-factor CFA model decreased monotonically when the amount of missing data increased. The RMSEA and SRMR statistics of Figure 3B mostly signified poor fit for the one-factor CFA model and always good fit for the other models. The statistics of the one-factor CFA model monotonically increased when the amount of missing data increased.

**Figure 4:**
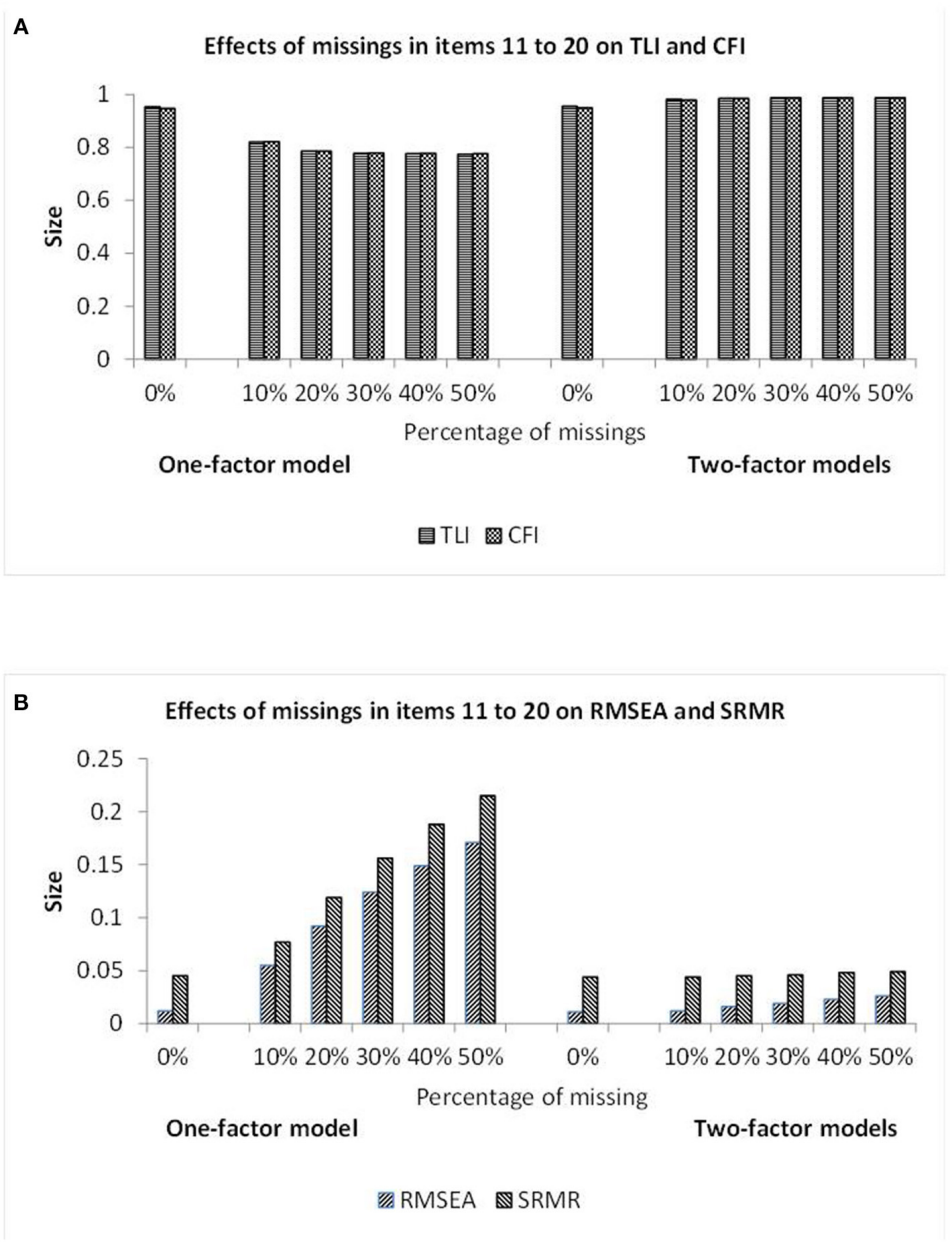
Illustration of the TLI and CFI results **(A)** and also the RMSEA and SRMR results **(B)** observed when investigating the data showing different percentages of missing observations in items 11–20 by one-factor (see first half) and two-factor (see second half) CFA models. Note: The missing data and semi-hierarchical CFA models yielded the virtually same results.

Figure 4 includes the fit results for datasets with missing data in 10 columns as bars. The bars representing the TLI, CFI, RMSEA, and SRMR statistics in Figures 4A,B signify poor model fit for the one-factor CFA model and good model fit for the other models. The RMSEA and SRMR statistics of the one-factor CFA model reflected the amount of missing data to a considerable degree whereas the TLI and CFI statistics appeared to show a floor effect after an initial decrease. The statistics for the other models appeared to be rather independent of the amount of missing data.

In sum, missing data did not impair model fit estimated by the missing data CFA model and the semi-hierarchical CFA model whereas there was impairment in investigations by the one-factor CFA model.

### Results Regarding Accuracy

Table 2 provides the mean standardized estimates of factor loadings for the models observed in investigating the datasets showing different percentages of missing data.

**Table 2:** Mean factor loadings obtained by the one-factor CFA model, the missing data CFA model and the semi-hierarchical CFA Model in investigating data showing different percentages of missing data (and percentages in parentheses) (*N*_withoutmissingdata_ = 500).

		**Factor loadings (percentages with respect to EV)^a^**					
		**One-factor CFA model**		**Missing data CFA model**		**Semi-hierarchical CFA model**	
**Item range^b^**	**Percentage of missing data**	**Mean**	**(Percent)**	**Mean**	**(Percent)**	**Mean**	**(Percent)**
17–20	10	0.357	(102)	0.347	(99)	0.350	(100)
	20	0.359	(103)	0.342	(98)	0.347	(99)
	30	0.360	(103)	0.338	(97)	0.346	(99)
	40	0.361	(103)	0.336	(96)	0.345	(99)
	50	0.360	(103)	0.333	(95)	0.345	(99)
14–20	10	0.382	(109)	0.338	(97)	0.342	(98)
	20	0.404	(115)	0.331	(95)	0.338	(97)
	30	0.423	(121)	0.327	(93)	0.336	(96)
	40	0.441	(126)	0.324	(92)	0.335	(96)
	50	0.457	(130)	0.320	(92)	0.335	(96)
11–20	10	0.431	(123)	0.327	(93)	0.333	(95)
	20	0.494	(141)	0.318	(91)	0.327	(93)
	30	0.548	(157)	0.312	(89)	0.324	(92)
	40	0.599	(171)	0.308	(88)	0.323	(92)
	50	0.644	(184)	0.304	(87)	0.323	(92)

a *The expected value (EV) of the factor loading is 0.35, and the percentage assigned to EV is 100*.

b *The items of this range show missing data*.

The expected value (EV) (see Figure 2) for the factor loadings was 0.35. In order to facilitate the reading of Table 2, we calculated percentages that were scaled to indicate the value of 100 in the case of correspondence of factor loading and EV and zero in the case of the value of 0. The percentages for the one-factor CFA models varied between 102 and 184, for the missing data CFA model between 99 and 87 and for the semi-hierarchical CFA model between 100 and 92. The deviations from EV indicated that overestimation characterized the factor loadings of the one-factor CFA model whereas underestimation was characteristic of the factor loadings of the other models. In all models the deviation increased when the number of columns including missing data was increased and also when the percentage of missing data was increased.

No deviation from EV was only observed for the semi-hierarchical CFA model when only four columns showed missing data and the percentage of missing data was 10%. A deviation of <5% was observed in virtually all investigations of datasets with missing data in four columns. Using the semi-hierarchical CFA model, a deviation of <5% was also found in investigations of datasets with missing data in seven out of 20 columns.

**Figure 5:**
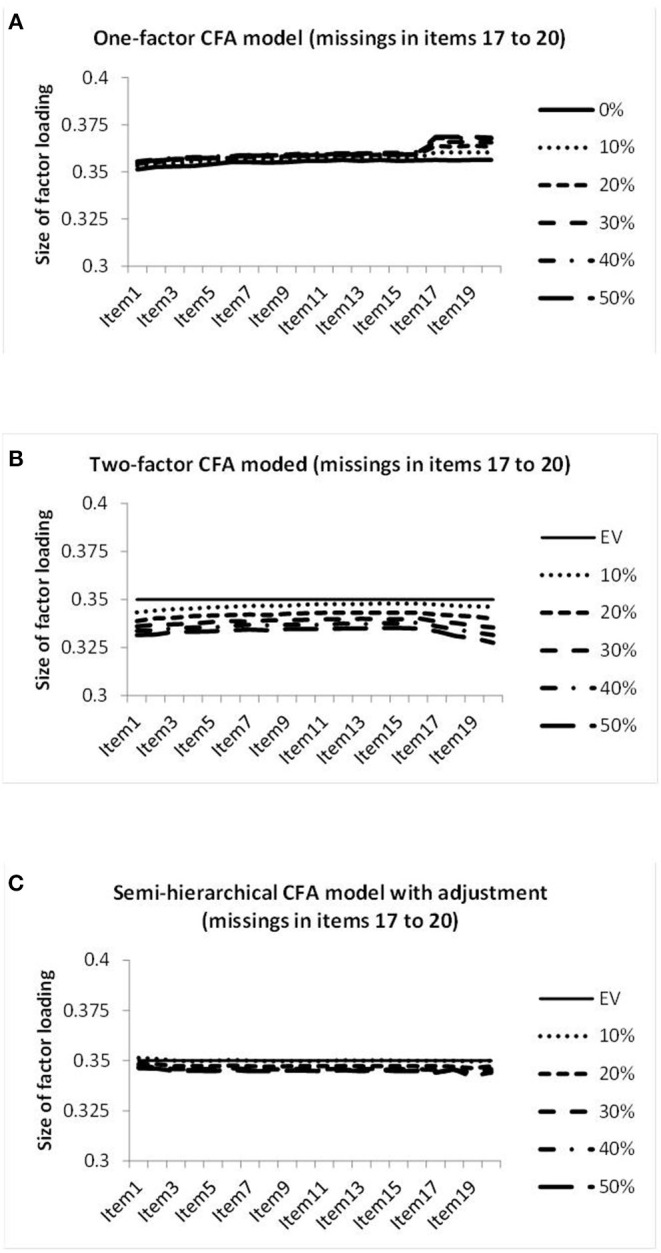
Illustration of the sizes of the factor loadings estimated by the one-factor CFA model **(A)**, the two-factor CFA model **(B)**, and the semi-hierarchical model **(C)** when investigating the data showing different percentages of missing observations in items 17–20.

In order to find out whether the deviations were the same for all columns or depended on the amount of missing data, we prepared graphical representations of the standardized estimates of factor loadings on the genuine factor as curves over the columns. Figure 5 provides the graphical representations of the factor loadings obtained by the three models in investigating the datasets with missing data in four columns.

Figure 5A reveals factor loadings of increased size in the columns showing missing data for the one-factor CFA model. Figure 5B shows factor loadings of decreasing size toward the end of the sequence of columns for the missing data CFA model. In contrast, all factor loadings of the semi-hierarchical CFA model displayed virtually the same size, as is obvious from Figure 5C. In the following we refer to this property as *size equivalence*. Factor loadings of the same size could also be characterized as consistent. But in test construction the term consistency already showed a specific meaning that was to be avoided.

**Figure 6:**
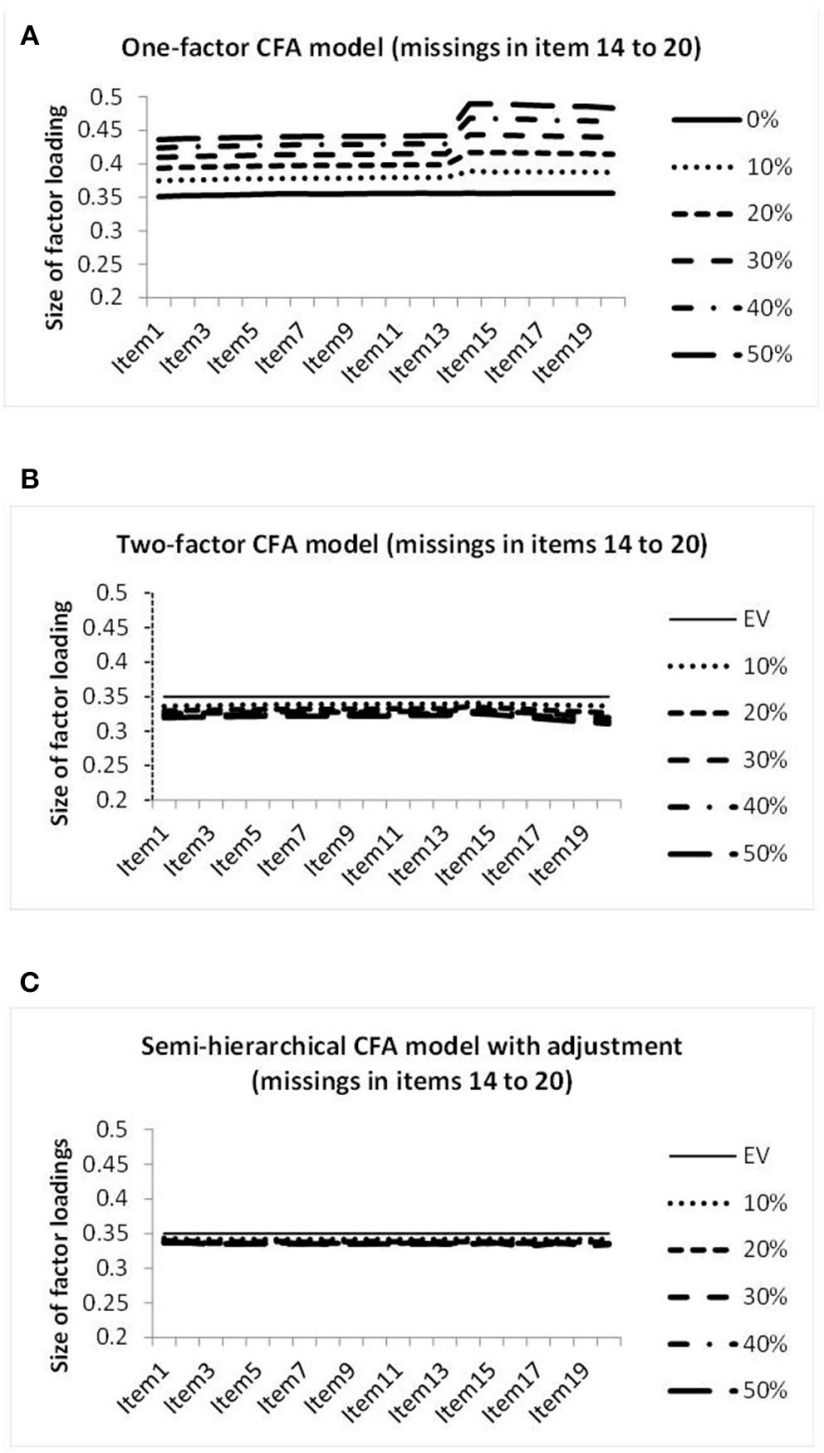
Illustration of the sizes of the factor loadings estimated by the one-factor CFA model **(A)**, the two-factor CFA model **(B)**, and the semi-hierarchical model **(C)** when investigating the data showing different percentages of missing observations in items 14–20.

The graphical representations of the factor loadings on the genuine factor achieved when investigating datasets with missing data in seven columns are included in Figure 6.

Figure 6 differs from Figure 5 according to the range of values covered by the vertical axis. The display reaches from 0.2 to 0.5 instead of from 0.3 to 0.4. Figure 6A reveals deviations in the columns showing missing data for the one-factor CFA model. The curves of Figure 6B for the missing data CFA model show a bit of decrease in the factor loadings in the beginning and in the end of the arrangement of columns. The decrease in the end is stronger and comparable to the decrease observed in the data with four columns showing missing data. The factor loadings depicted in Figure 6C for the semi-hierarchical CFA model are virtually size-equivalent.

**Figure 7:**
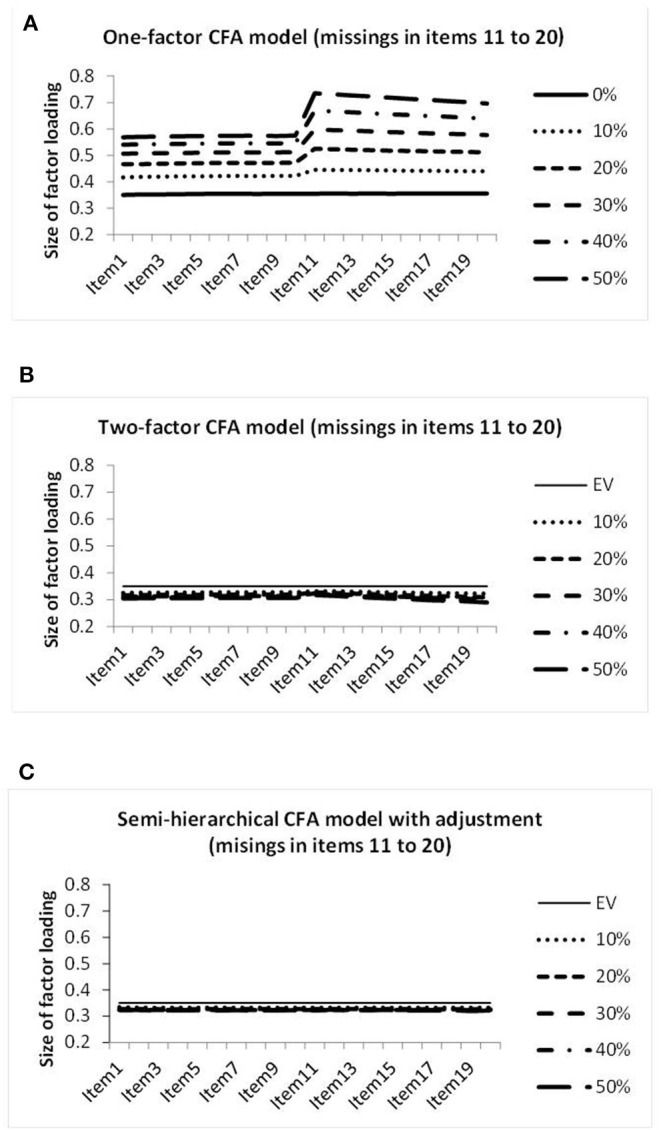
Illustration of the sizes of the factor loadings estimated by the one-factor CFA model **(A)**, the two-factor CFA model **(B)**, and the semi-hierarchical model **(C)** when investigating the data showing different percentages of missing observations in items 11 to 20.

Missing data in 10 columns led to the factor loadings on the genuine factor illustrated in Figure 7. Because of the considerable increase in the deviation of the factor loadings observed by the one-factor CFA model, the range of the vertical axis is again increased. It reaches from 0.2 to 0.8. Figure 7A reveals large increases in the factor loadings of the columns with missing data for this model. Figure 7B seems to reveal a very minor deviation from size equivalence for the missing data CFA model, and Figure 7C reveals virtually perfect size equivalence for the semi-hierarchical CFA model.

Because of the great importance of correspondence of the factor loadings for columns without missing data and columns with missing data, standardized absolute differences between the average standardized estimates of factor loadings for columns showing no missing data and for columns with missing data were computed and included in Table 3.

**Table 3:** Standardized absolute differences between the mean factor loadings for items without missing data and items with missing data obtained by the one-factor CFA model, the missing data CFA model and the semi-hierarchical CFA model in investigating data showing different percentages of missing data (*N*_withoutmissingdata_ = 500).

	**Standardized absolute differences**			
**Item range^a^**	**Percentage of missing data**	**One-factor CFA model**	**Missing data CFA model**	**Semi-hierarchical CFA model**
17–20	10	2.60	0.12	0.14
	20	3.99	0.54	0.30
	30	5.05	1.17	0.54
	40	6.09	1.81	0.82
	50	6.92	2.42	0.73
14–20	10	6.79	0.03	0.01
	20	12.86	0.53	0.22
	30	19.20	1.01	0.35
	40	25.48	1.44	0.42
	50	31.82	1.91	0.53
11–20	10	14.70	0.02	0.11
	20	32.17	0.48	0.19
	30	52.21	0.93	0.28
	40	74.17	1.24	0.40
	50	96.57	1.73	0.05

a *The items of this range show missing data*.

Standardization of the difference was achieved by dividing the absolute difference of interest by the absolute difference that was observed for the one-factor CFA model when applied to complete data. Standardized absolute differences smaller or equal to one were considered as good.

The inspection of Table 3 reveals that the one-factor CFA model did not even lead to one good result. In the missing data CFA model seven out of 15 statistics were good and in the semi-hierarchical CFA model all statistics were good.

In sum, the factor loadings of the one-factor model showed increased average sizes, and the factor loadings of the other models showed decreased average sizes. Furthermore, the factor loadings of the one-factor model displayed large variation due to missing data whereas the factor loadings of the missing data model only showed minor variation and size equivalence characterized the factor loadings of the semi-hierarchical model.

## Discussion

One major problem with missing data is that we never know what would have been the responses that did not occur in the past and not found their way into the dataset. We can speculate about the missing responses. We can search the context and the population for clues and consider the type of missing data (e.g., Little and Rubin, 2019). We can make assumptions based on psychological theories, and we can employ the other information obtained from each specific participant since the concept of missing data implies that some information about the participant is available (Vogt and Johnson, 2015). Whatever we propose as a replacement, we can never be sure whether the response would have turned out in this way in the past.

Incompleteness of a dataset can be overcome by methods developed for the replacement of missing data. Values for replacing missing data can, for example, be estimated within the maximum likelihood framework (Enders, 2013) or created by multiple imputation (van Buuren, 2018). However, including additional information into a dataset is not without consequences for this dataset. Especially, the additional information can increase the consistency among the items and, thus, increase the probability of detecting something that appears to underlie the data. If there is a larger percentage of missing data, replacement can improve the consistency of the dataset quite a bit. It can even create the impression of an underlying dimension where there was no such dimension before.

For avoiding any kind of influence on the properties of the dataset, our study explores the possibility of investigating the dataset without any replacement of missing data. In this point there is similarity with the approach suggesting the use of planned missing data designs that are proposed for longitudinal research. Planned missing data designs are incomplete designs; they omit data that are not really necessary for valid statistical investigations. The focus of these investigations is on effects and relationships but not on the structural properties of the data, as in our study.

The success of the modeling approach depends on its suitability for the missingness characterizing the dataset. Our study employed two ways of modeling missing data that could be considered suitable for incomplete datasets. The first way is reflected by the missing data CFA model. It assumes two sources of systematic variation that are captured by two latent variables. These latent variables are assumed to contribute simultaneously to the manifest variables. One of the two latent variables is focused on the systematic variation due to missing data that can be perceived as method effect. Method effects are closely linked to characteristics of measurement (Maul, 2013; Schweizer, 2020). In this case it is the design of measurement. The other way is represented by the semi-hierarchical CFA model. It additionally reflects the special relationship of the two sources of systematic variation. One source is assumed to determine the quality of the response as correct or incorrect and the other source whether a response is given. The semi-hierarchical CFA model reflects this special relationship by integrating it into a hierarchical structure that is characteristic of a sample composed of two different subsamples (Raudenbush and Bryk, 2002; Snijders and Bosker, 2012).

The study compares these models among each other and with the one-factor CFA model regarding model fit and accuracy in parameter estimation. The results concerning model fit are good for the missing data CFA model and the semi-hierarchical CFA model, irrespective of the missing data condition. The fit statistics observed by these models were even slightly better than those for the one-factor CFA model when applied to complete data. This minor effect is presumably due to the difference between variation originating from missing data and the mixture of variation originating from the genuine source and random influences. When arranged as a regular array, variation originating from missing data can be expected to be more systematic, i.e., it leads to the larger explained variance. The missing data CFA model and the semi-hierarchical CFA model performed equally well although the semi-hierarchical CFA model was supposed to perform slightly better (Schweizer et al., 2020a). This lack of difference was presumably due to not enough variability in the sizes of the subsamples of the generated datasets.

The investigation of the accuracy of the estimates of the factor loadings yielded different results for the models. An important part of this investigation was the comparison of the sizes of the factor loading estimates for the columns in order to evaluate size equivalence. Using the one-factor CFA model, the differences between the factor loading estimates for the two types of columns were always large. In the missing data CFA model there were small deviations from size equivalence. In contrast, the estimates of factor loadings achieved by the semi-hierarchical CFA model showed virtually perfect size equivalence.

The effect of the amount of missing data was most obvious in the average sizes of the factor loadings. The larger the number of incomplete columns and the larger the percentage of missing data, the larger was the average size of the factor loadings in investigations using the one-factor CFA model whereas in the other models the average size was smaller. The largest absolute deviation was always observed for the one-factor CFA model and the smallest absolute deviation was always observed for the semi-hierarchical CFA model.

Although the investigation of the accuracy of the factor loadings revealed size equivalence of the estimates for the semi-hierarchical CFA model that performed best regarding accuracy, the outcomes regarding the average size of the factor loadings were less than optimal even for this model: the average size stayed below the expected value and the deviation showed dependency on the number of incomplete columns and the percentage of missing data. Despite these deviations from expectations, the results of this study demonstrate advantages of the modeling of missing data, as is specified in detail in the following paragraphs.

First, since the investigation of model fit is a check of the assumption regarding the structure of data in the first place, exact correspondence of expected and estimated factor loadings is not required. The missing data CFA model and the semi-hierarchical CFA model serve the investigation of the assumption that one general latent variable representing the construct of interest and a specific missing data latent variable are underlying the data. The observation of good model fit by these models suggests that this assumption holds for the investigated dataset. This is what a researcher normally likes to learn when investigating the structure of data. The researcher notices that the one-factor model does not explain the data well in the first step. The missing data are the obvious reason for the lack of good model fit. Therefore, the researcher investigates the structure of the data by models that take the missing data as possible source of the misfit into consideration in the second step. The positive outcome of the investigation tells the researcher that the failure in the first step was actually due to the missing data and that the structural assumption was correct.

Second, if underestimates of factor loadings, as can be observed by the semi-hierarchical CFA model, satisfy the other requirements for factor loadings, accepting them as basis for decisions regarding the suitability of items does not imply the danger of retaining invalid items. Third, it is a constant that relates the observed factor loadings to the expected value; it can be perceived as a component of a linear model. It should be possible to estimate this component in a Monte Carlo Simulation study (Paxton et al., 2001) if necessary and to adjust the factor loadings appropriately.

Finally, we like to point out that there are some limitations of the reported study. First, the results extend to binary data only. Second, although we think that the size of the investigated datasets is similar to many datasets that are investigated in empirical research, the lack of the variation of the size may be perceived as a shortcoming. Third, there is no variation of the expected value of the factor loadings. However, despite these limitations, it was possible to demonstrate that valuable results are achievable in structural investigations of incomplete data without any replacement of missing data.

## Data Availability Statement

The datasets presented in this article are not readily available because only the starting numbers were saved, not the generated data. The starting numbers of the generated data sets can be made available to fully reproduce the data. Requests to access the datasets should be directed to Karl Schweizer, k.schweizer@psych.uni-frankfurt.de.

## Author Contributions

KS conceptualized the study and contributed to the writing. DK contributed substantially to the writing and the manuscript management. AG and TW contributed substantially to the writing. All authors contributed to the article and approved the submitted version.

## Conflict of Interest

The authors declare that the research was conducted in the absence of any commercial or financial relationships that could be construed as a potential conflict of interest.
